# Proteomic profiling reveals dynamic regulation of vesicle trafficking across glioma grades

**DOI:** 10.1007/s11060-025-05151-5

**Published:** 2025-07-24

**Authors:** Tomasz Pienkowski, Patrycja Mojsak, Tomasz Kowalczyk, Dominik Cysewski, Mikolaj Krupa, Robert Rutkowski, Zenon Mariak, Adrian Godlewski, Joanna Reszec, Marcin Moniuszko, Adam Kretowski, Tomasz Lyson, Michal Ciborowski

**Affiliations:** 1 Metabolomics and Proteomics Laboratory, Clinical Research Centre, Medical University of Bialystok, M. Sklodowskiej-Curie 24A, Bialystok, 15-276 Poland; 2 Department of Neurosugery, Medical University of Bialystok, Bialystok, 15-276 Poland; 3 Department of Medical Pathomorphology, Medical University of Bialystok, Bialystok, 15-269 Poland; 4 Department of Regenerative Medicine and Immune Regulation, Medical University of Bialystok, Bialystok, 15-269 Poland; 5 Department of Allergology and Internal Medicine, Medical University of Bialystok, Bialystok, 15-276 Poland; 6 Department of Endocrinology, Diabetology and Internal Medicine, Medical University of Bialystok, Bialystok, 15-276 Poland; 7 Department of Interventional Neurology, Medical University of Bialystok, Bialystok, 15-276 Poland; 8 Department of Medical Biochemistry, Medical University of Bialystok, 15-089, Bialystok, Poland

**Keywords:** GBM, Glioma, Proteomics, LC-MS, TMT-labeling

## Abstract

**Purpose:**

Gliomas are highly heterogeneous central nervous system tumors that evolve through progressive molecular reprogramming. While cell proliferation and adhesion mechanisms are well-characterized, the contribution of vesicle trafficking to glioma progression remains underexplored. This study aimed to characterize proteomic changes across glioma grades.

**Methods:**

We performed untargeted, quantitative proteomic profiling of glioma tissues across WHO grades I–IV using a combination of Tandem Mass Tag (TMT)-11plex labeling and high-resolution liquid chromatography–mass spectrometry (LC-MS). Tissue samples were processed using filter-aided sample preparation (FASP) and analyzed using a µPAC reverse-phase HPLC system coupled to a high-resolution mass spectrometer. Protein identification and quantification were conducted through database searching and validated against stringent quality control criteria.

**Results:**

We identified over 4,400 proteins across samples, revealing dynamic, grade-specific shifts in vesicle trafficking. Grade II gliomas showed upregulation of exocytic proteins (e.g., synaptotagmin, syntaxin, clathrin) and suppression of dynamin, suggesting enhanced vesicular secretion. Grade III tumors exhibited the opposite trend—marked downregulation of exocytic components with concurrent activation of clathrin-mediated endocytosis. Grade IV gliomas displayed a hybrid profile, with partial reactivation of exocytic machinery alongside sustained endocytic activity, indicative of vesicular plasticity.

**Conclusion:**

This study highlights the synaptic vesicle cycle as a progressively remodeled pathway in glioma biology. Our findings suggest that vesicle trafficking is a critical, underrecognized feature of glioma pathogenesis and may represent a novel axis for therapeutic exploration.

**Supplementary Information:**

The online version contains supplementary material available at 10.1007/s11060-025-05151-5.

## Introduction

Gliomas are the most common primary malignant tumors of the central nervous system (CNS), originating from glial cells that provide structural and metabolic support to neurons [1]. They affect both children and adults and can arise in the brain or spinal cord [2]. Gliomas are characterized by their diffuse infiltration into healthy neural tissue, which complicates both diagnosis and treatment. In adults, the incidence ranges from 1.9 to 9.6 per 100,000 individuals per year, depending on age, sex, ethnicity, and geographic region [3]. Clinically, gliomas present with a range of neurological symptoms such as headaches, seizures, cognitive decline, or motor deficits, depending on tumor location and size.

Current standard treatment consists of maximal safe surgical resection, followed by post-surgical therapy. However, the effectiveness of these treatments is hindered by the diffuse infiltration of gliomas and the absence of well-defined tumor margins [4]. Despite advancements in neuroimaging, accurately distinguishing glioma subtypes preoperatively remains challenging, with definitive diagnosis typically relying on histopathological analysis of resected tissue [5]. Gliomas are histologically classified into several subtypes—including astrocytomas, oligodendrogliomas, and ependymomas—and graded by the World Health Organization (WHO) on a scale from 1 to 4, reflecting increasing malignancy. Low-grade gliomas (grades 1–2) typically grow more slowly and may remain stable for extended periods, while high-grade gliomas (grades 3–4) are aggressive, highly invasive, and associated with a poor prognosis [2]. Despite histological differences, all gliomas pose a significant therapeutic challenge due to their tendency to invade surrounding brain tissue, making complete surgical resection nearly impossible and contributing to high recurrence rates [6].

Recent studies have emphasized the role of glioma plasticity in driving disease progression, therapy resistance, and immune evasion [7, 8]. Tumor plasticity refers to the dynamic ability of cancer cells to adapt to microenvironmental pressures by modulating their phenotype, gene expression, and behavior. In gliomas, this plasticity manifests through transitions between proliferative and invasive cellular states, acquisition of stem-like features, metabolic reprogramming, and remodeling of the tumor microenvironment. Several studies have demonstrated that glioma cells can hijack neuronal signaling pathways to promote their own growth and infiltration, a process that underscores the functional integration of glioma into neural circuits [9–11]. For instance, Venkataramani et al. [10] showed that glioma cells form functional synapses with neurons and use glutamate signaling to enhance their proliferation. Zeng et al. [11] reported that gliomas engage in synapse-like communication with surrounding neurons via AMPA receptors. These findings suggest that glioma plasticity is closely tied to neurosecretory mechanisms, yet the molecular dynamics of these processes across tumor grades remain incompletely understood.

Despite this emerging knowledge, prior studies have not systematically examined how global proteomic reprogramming related to vesicular trafficking evolves across different glioma grades. Moreover, nonlinear, stage-specific changes in protein expression that may reflect dynamic adaptation strategies have received little attention. The majority of molecular studies in glioma progression focus on monotonic trends in gene expression or epigenetics, with limited exploration of global protein-level regulation in the context of tumor plasticity [12–15].

To better understand the molecular underpinnings of glioma progression, we performed an unbiased, global proteomic analysis across different glioma grades. This revealed extensive reprogramming of cellular pathways, particularly those related to synaptic vesicle trafficking and exocytosis, components linked to neurotransmission. Interestingly, the expression patterns of several proteins did not follow a linear trajectory with tumor grade progression. Instead, some proteins exhibit nonlinear, grade-dependent abundance changes. These patterns may reflect the dynamic functional demands of glioma cells at different stages, as they adapt to, and potentially influence, the surrounding neural microenvironment contributing to tumor plasticity.

Although not the initial focus of our investigation, this vesicular remodeling emerged as a critical feature that aligns closely with recent literature implicating glioma-driven neurotransmitter secretion in shaping the peritumoral microenvironment and facilitating tumor–neuron crosstalk [16]. The progressive modulation of vesicle cycle components we observed across glioma grades suggests that glioma cells adapt their secretory machinery dynamically to meet stage-specific demands, supporting oncogenic signaling, metabolic adaptation, and immune modulation. This study provides new insights into glioma plasticity by integrating global proteomic data with established neurosecretory mechanisms, highlighting vesicular trafficking as a potential therapeutic vulnerability in glioma progression.

## Materials and methods

### Study design

This study analyzed a consecutive series of glioma tissue samples obtained from patients who underwent neurosurgical procedures at the Department of Neurosurgery, University Hospital of Bialystok, between 2016 and 2021. All samples were collected and stored in compliance with the Biobank standards of the Medical University of Bialystok [17]. Prior to surgery, each patient received standard neurosurgical care, including a two-day course of corticosteroids to reduce peritumoral edema. All participants provided informed consent before sample collection. Procedures followed institutional guidelines at Bialystok University Hospital and Good Clinical Practice, in accordance with the Declaration of Helsinki. The study used archival material from the Biobank of the Medical University of Bialystok, Poland [18].

Histopathological classification of the tumor specimens was performed according to the fourth edition of the World Health Organization (WHO) Classification of Tumors of the Central Nervous System (CNS4th), which was current at the time of tissue collection [19]. Based on this classification, the glioma samples were astrocytomas stratified into five groups: WHO Grade 1 (G1, *n* = 7), Grade 2 (G2, *n* = 10), Grade 3 (G3, *n* = 10), Grade 4 IDH-WT (G4-WT, *n* = 11), and Grade 4 IDH-MT (G4-MT, *n* = 11). Grades from 1 to 3 were IDH-WT (S. Table 1).

To ensure data quality and consistency, tissue samples were excluded if they were derived from pediatric patients, recurrent tumors, or exhibited poor preservation quality. Additional exclusion criteria included prior oncological treatment, significant comorbidities, known genetic syndromes, or incomplete clinical information.

The group selection was based on a prior study that utilized serum samples from the same cohort of patients [20], ensuring consistency between tissue- and serum-based investigations.

### Sample preparation

#### Proteomics analysis

Tissue specimens were processed on ice immediately after retrieval. Each sample was divided into two portions: one designated for histopathological examination and the other for proteomic analysis. Proteomic samples were rapidly snap-frozen in liquid nitrogen (− 195.8 °C) within 5 min of excision and subsequently stored at − 80 °C to preserve protein integrity.

For proteomic preparation, tumor tissues of various WHO grades were first pulverized under liquid nitrogen and then lysed on ice using a buffer containing 5% sodium deoxycholate (SDC), one PhosSTOP tablet (Roche), one cOmplete™ protease inhibitor cocktail tablet (Roche), 50 mM tris(2-carboxyethyl)phosphine (TCEP), and 50 mM triethylammonium bicarbonate (TEAB). Protein reduction was performed at 60 °C for 30 min, followed by alkylation at room temperature using 15 mM iodoacetamide (IAA) in 50 mM TEAB. Total protein concentration was quantified using the bicinchoninic acid (BCA) assay.

Protein digestion was conducted using 30 kDa Microcon^®^ centrifugal filters, applying the Filter-Aided Sample Preparation (FASP) method. Proteins were digested overnight at 37 °C with a Trypsin/LysC mix at a 1:50 enzyme-to-protein ratio in 100 mM TEAB. For quantification and multiplexing, 50 µg of peptides were labeled with Tandem Mass Tag (TMT) reagents according to the manufacturer’s instructions. Labeling was carried out at room temperature for 1 h, followed by quenching with 5% quenching reagent for 15 min. To stop residual protease activity, 0.5% trifluoroacetic acid (TFA) was added. SDC was removed using ethyl acetate phase separation and two washing steps. Labeled peptides were desalted using Pierce™ C18 Tips and fractionated into eight fractions with the Pierce™ High pH Reversed-Phase Peptide Fractionation Kit. The resulting fractions were dried under vacuum using a SpeedVac concentrator in preparation for LC-MS/MS analysis.

**Fig. 1:**
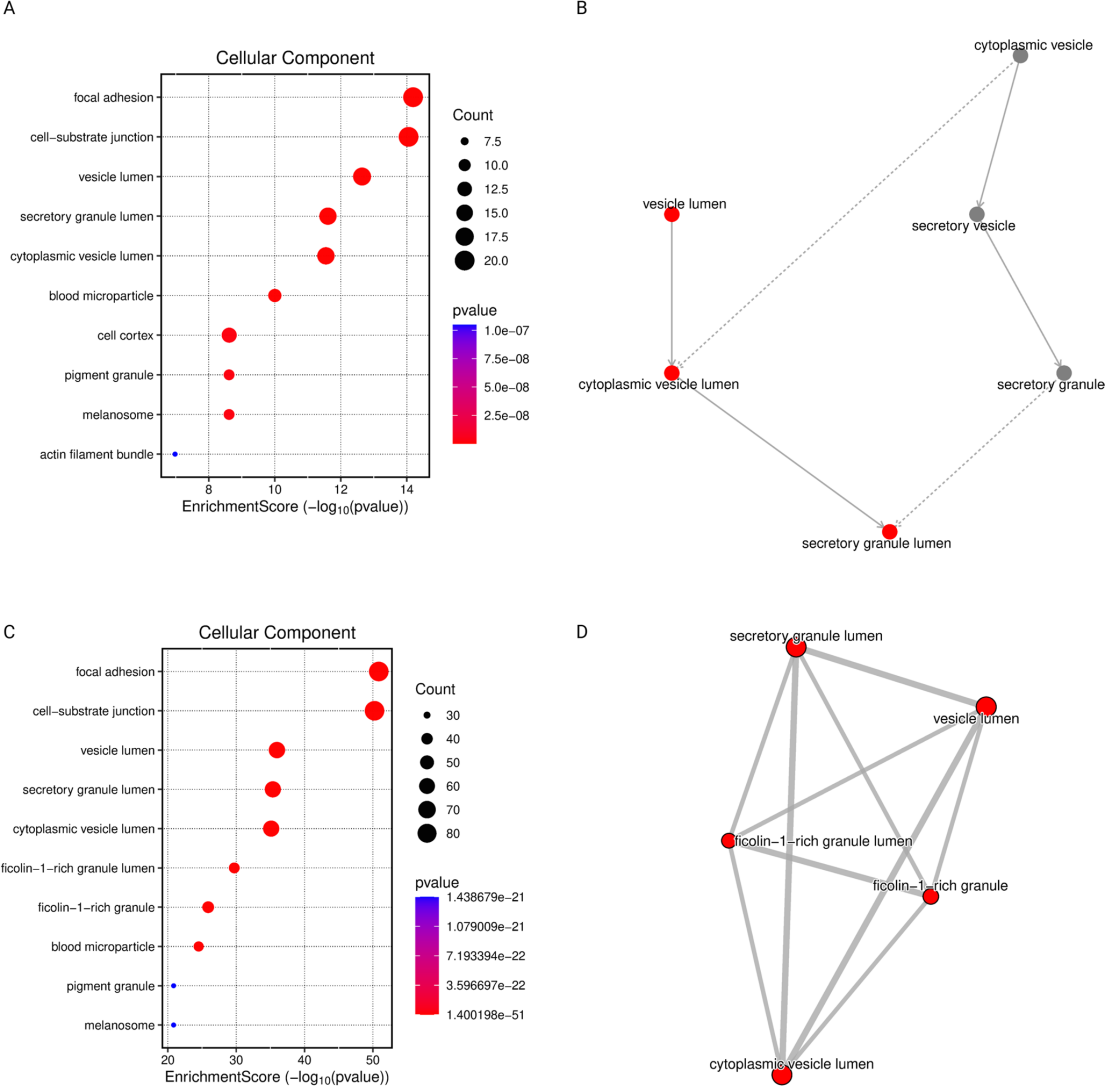
Gene Ontology-Based Functional Network Comparing IDH-WT vs. IDH-MT WHO Grade 4 Gliomas and IDH-WT Grade 4 vs. Grade 3 Gliomas. (**A**) The dot plot displays enriched GO terms of comparison between who grade 4 IDH-WT and who grade 4 IDH-MT gliomas, where dot size represents the number of genes per term, and color intensity reflects statistical significance (p-value). (**B**) Gene Ontology enrichment network graph of IDH-WT vs. IDH-MT WHO Grade 4 Gliomas comparison. (**C**) The dot plot displays enriched GO terms of comparison between WHO Grade 4 IDH-WT and WHO Grade 3 gliomas, where dot size represents the number of genes per term, and color intensity reflects statistical significance (p-value). (**B**) Gene Ontology enrichment network graph of WHO Grade 4 and WHO Grade 3 Gliomas comparison. Gene Ontology terms are represented as nodes and are interconnected by edges that denote functional similarity or gene set overlap. Solid lines represent direct functional or hierarchical relationships, while dotted lines indicate weaker or indirect associations. The width of each edge is proportional to the strength of the association, with thicker lines indicating a higher degree of similarity (greater gene overlap or semantic similarity).

To ensure accurate relative quantification and minimize inter-batch variability, a TMT-11plex labeling strategy was employed. Each set included 10 biological samples (tumor tissues of different glioma grades) and one internal standard (IS). The IS was prepared by pooling equal protein amounts (10 µg) from all included samples, and labeled consistently with TMT channel 126 across all sets. Experimental samples were assigned to the remaining TMT channels (127 N–131 C) (S. Figure 1). In total, 5 TMT-11plex sets were processed, covering 49 unique tumor tissue samples. All samples were biological replicates; no technical replicates or external healthy tissue controls were included. Instead, internal standard–based normalization was applied both within and across batches to support relative protein quantification between different molecular glioma subtypes. Due to the inherent limitation of the TMT-11plex system in sample capacity, we employed a cross-set normalization strategy using the IS channel (126) as a common reference point across all sets. This approach enabled consistent quantification of relative protein abundance across the entire sample cohort.

Labeled peptide fractions were analyzed using the Dionex UltiMate 3000 RSLCnano system (Thermo Scientific) coupled with a reverse-phase high-performance liquid chromatography (RP-HPLC) setup using a trap-elute configuration. The analytical separation was performed using a 50 cm µPAC column (PharmaFluidics), featuring a lithographically etched C18 stationary phase, in conjunction with an Acclaim PepMap 100 C18 trap column (Thermo Scientific). Both columns were maintained at 45 °C throughout the run. The µPAC column was equilibrated in 95% mobile phase A (H₂O + 0.2% formic acid) and 5% mobile phase B (90% acetonitrile + 0.2% formic acid in H₂O), with a constant flow rate of 300 nL/min. The peptide elution gradient spanned 140 min: 3–23% B over 90 min, 23–40% B over 20 min, 40–95% B over 10 min, followed by a 5-minute hold at 95% B.

### Data treatment and statistical analysis

Spectral identification and preliminary statistical analyses were carried out using PEAKS Studio version 11 (Bioinformatics Solutions Inc., Canada). Ion searches were performed with a precursor ion mass tolerance of 15 ppm and a product ion tolerance of 0.5 Da. Carbamidomethylation of cysteine residues (+ 57.021 Da) was set as a fixed modification, while oxidation of methionine (+ 15.995 Da) and deamination of amino acids (+ 0.98 Da) were treated as variable modifications. To mitigate the impact of potential contaminants—an acknowledged challenge in proteomics—we employed a dedicated contamination database to identify and remove non-specific features from the dataset.

To ensure the reliability of peptide identifications, both a decoy (“reverse”) sequence database and a randomized database were utilized for validation. Protein identification required a minimum of one sequence-unique peptide and a peptide-level False Discovery Rate (FDR) threshold of 1%.

Statistical analyses were performed using the Perseus platform. A multi-sample ANOVA was applied to detect proteins significantly altered across glioma grades, using a permutation-based FDR threshold of 0.05. Significant proteins were further filtered using FDR-adjusted p-values (q < 0.05).

Subsequent analyses were conducted using Ingenuity Pathway Analysis (IPA, Qiagen). z-scores were used to evaluate the relative abundance of proteins, enabling the identification of significant alterations across glioma grades. The IPA Core Analysis module was employed to explore perturbed biochemical pathways, upstream regulators, regulatory mechanisms, molecular functions, and potential protein-protein interactions. Pathway-level significance was assessed using z-scores (with thresholds of ≤–2 or ≥ 2 indicating inhibition or activation, respectively) and Benjamini–Hochberg adjusted p-values.

### Pathway enrichement analysis

To further support comprehensive pathway interpretation and data visualization, a suite of open-source tools was utilized. SRplot, a web-based platform, was used for generating high-quality customizable graphs crossing proteomic data with KEGG pathway databases. Functional enrichment analysis and biological term classification were performed using the clusterProfiler R package, which enabled systematic comparison of protein clusters to uncover dominant biological themes [21]. In parallel, Pathview, an R/Bioconductor tool, was used to map data onto KEGG pathway diagrams, offering intuitive visualizations while supporting diverse data types and species [22].

To ensure an unbiased and comprehensive exploration of biologically relevant alterations, pathway analyses were performed without restrictive filters for tissue type, disease state, or biological fluid. This approach was chosen to capture both canonical and non-canonical pathways potentially involved in glioma progression and prognosis, while allowing for subsequent biological interpretation and validation.

## Results

In total, 2,532, 2,378, 2,645, 2,036, and 2,155 proteins were identified in TMT Sets 1 through 5, respectively. These values represent protein identifications within individual 11plex batches and are lower than the totals reported for group-wise comparisons, which reflect merged data across multiple sets. The higher counts in group-level analyses result from combining biological replicates, allowing proteins quantified in only a subset of samples to be retained in the final comparison matrices.

**Fig. 2:**
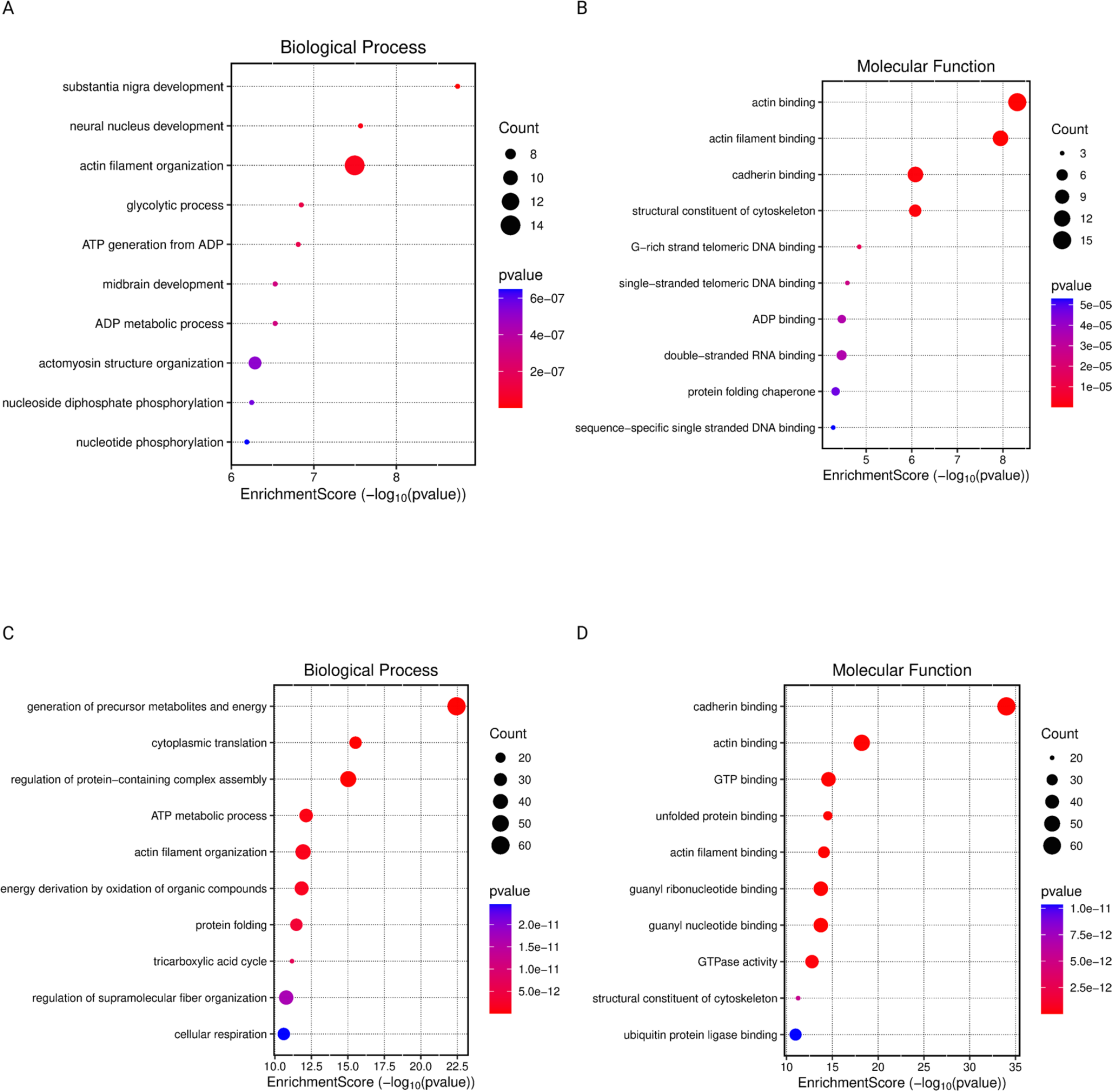
Gene Ontology-Based analysis of Biological Processes and Molecular Functions Comparing IDH-WT vs. IDH-MT WHO Grade 4 and WHO Grade 4 vs. Grade 3 Gliomas. (**A**) Dot plot of enriched GO Biological Processes comparing IDH-WT vs. IDH-MT WHO Grade 4 gliomas. Dot size represents the number of genes associated with each term, and color intensity reflects statistical significance (p-value); (**B**) Dot plot of enriched GO Molecular Functions comparing IDH-WT vs. IDH-MT WHO Grade 4 gliomas. Dot size represents the number of genes associated with each term, and color intensity reflects statistical significance (p-value); (**C**) Dot plot of enriched GO Biological Processes comparing WHO Grade 4 vs. Grade 3 gliomas. Dot size represents the number of genes associated with each term, and color intensity reflects statistical significance (p-value); (**D**) Dot plot of enriched GO Molecular Functions comparing WHO Grade 4 vs. Grade 3 gliomas. Dot size represents the number of genes associated with each term, and color intensity reflects statistical significance (p-value)

Comparative proteomic analysis revealed that 119 proteins significantly distinguished G4-WT from G4-MT, with 105 proteins downregulated and 14 upregulated in G4-WT (S. Table 2). When comparing G4-WT to G3, 974 proteins exhibited differential expression, including 596 downregulated and 378 upregulated in G4-WT (S. Table 4). Between G3 and G2, 1,869 proteins were differentially expressed, with 733 downregulated and 1,136 upregulated in G3 (S. Table 6). Heat maps and volcano plots are available in supplementary materials (S. Figure 2). The total number of proteins reported refers to unique protein groups identified in the dataset, which may include multiple peptides, isoforms or distinct UniProt entries mapping to the same gene; therefore, the number of unique gene symbols is slightly lower due to redundancy inherent in proteomic datasets: 102 for the G4 IDH-WT vs. G4 IDH-MT comparison (S. Table 3), 542 for G4 IDH-WT vs. G3 (S. Table 5), 809 for G3 vs. G2 (S. Table 7), and 756 for G2 vs. G1 (S. Table 9).

Finally, comparison between G2 and G1 identified 1,544 differentially expressed proteins, with 970 downregulated and 574 upregulated in G2 (S. Table 8). Further characterization of unique proteins identified in each comparison revealed 4 proteins in the G4-WT versus G4-MT comparison (IGHA1, DCLK1, EPHX1, HEBP1), 41 proteins in the G4-WT versus G3 comparison, 61 proteins in the G3 versus G2 comparison, and 72 proteins in the G2 versus G1 comparison (S. Table 10).

We identified a total of 4,444 in G4-WT (S. Table 11), 4,275 in G4-MT (S. Table 13), 4,400 in G3 (S. Table 15), 3,957 in G2 (S. Table 17), and 4,275 proteins in G1 (S. Table 19). The total number of gene symbols: 1,479 in G4-WT (S. Table 12), 1,481 in G4-MT (S. Table 14), 1,470 in G3 (S. Table 16), 1.400 in G2 (S. Table 18), and 1,448 proteins in G1 (S. Table 20).

### Pathway entichement analysis

Gene Ontology (GO) enrichment analysis was conducted on the entire dataset without applying a fold-change threshold. In the comparison between G4-WT and G4-MT, the most significantly enriched biological processes were associated with substantia nigra development and neural nucleus development, but actin filament organization showed the higher gene count. The most enriched cellular components included focal adhesions, cell-substrate junctions, as well as vesicular, secretory, and cytoplasmic lumens. The predominant molecular functions identified were actin binding and cadherin binding (Figure 1AB) (Figure 2AB).

In contrast, the comparison between G4-WT and G3 revealed a different biological signature. The most significantly enriched biological process was the generation of precursor metabolites and energy. Notably, the enriched cellular components overlapped with those observed in the G4-WT vs. G4-MT comparison, indicating a conserved alteration in subcellular structural organization. However, the distribution of molecular functions differed; cadherin binding exhibited approximately twice the level of enrichment compared to actin binding. This contrasts with the G4-WT vs. G4-MT comparison, where the difference in enrichment between cadherin and actin binding was minimal (Figure 1CD) (Figure 2CD).

**Fig. 3:**
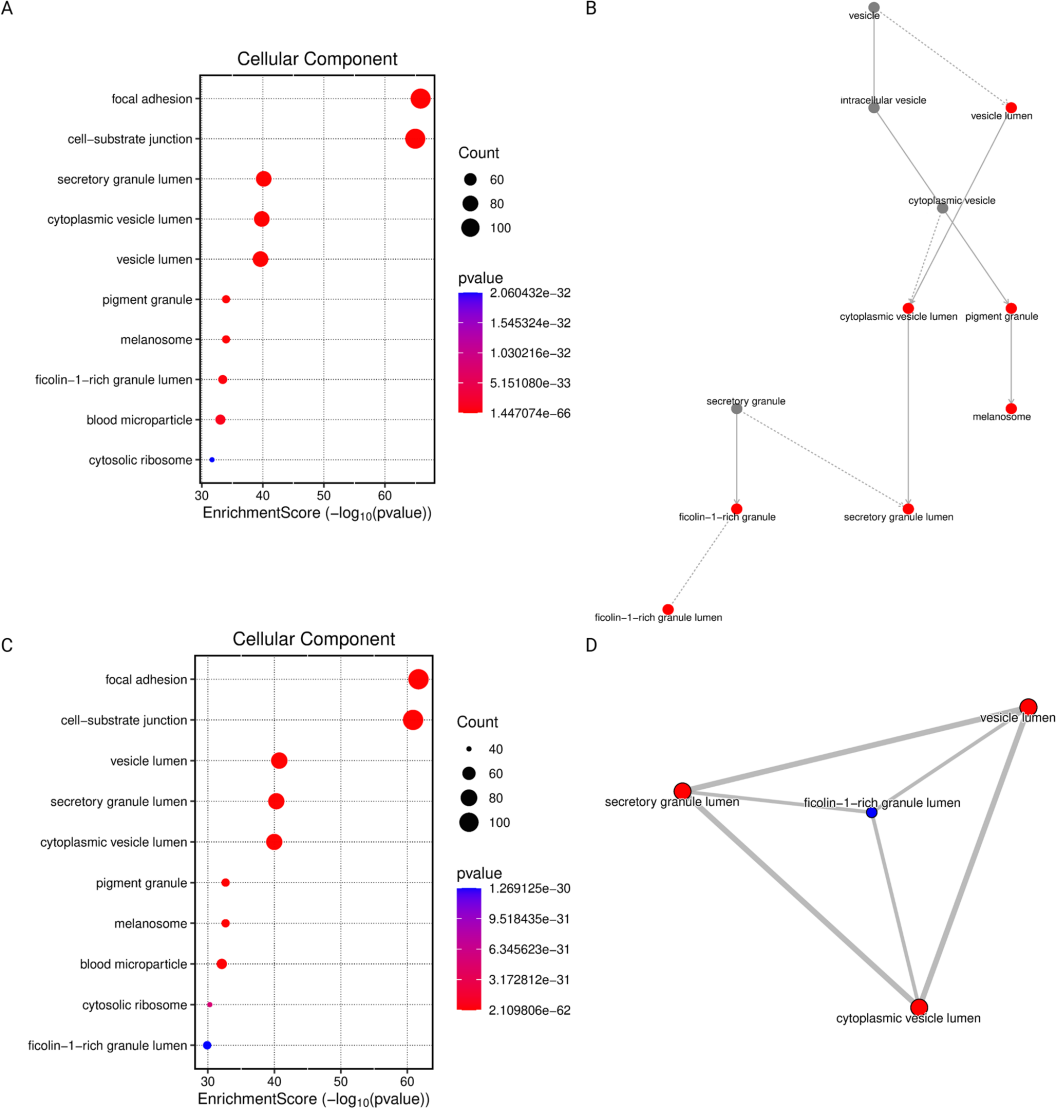
Gene Ontology-Based Functional Network Comparing WHO Grade 3 vs. Grade 2 and WHO Grade 2 vs. Grade 1 Gliomas. (**A**) Dot plot displaying enriched GO terms comparing WHO Grade 3 and Grade 2 gliomas. Dot size represents the number of genes associated with each term, and color intensity reflects statistical significance (p-value); (**B**) Gene Ontology enrichment network graph of the comparison between WHO Grade 3 and Grade 2 gliomas; (**C**) Dot plot displaying enriched GO terms comparing WHO Grade 2 and Grade 1 gliomas. Dot size represents the number of genes per term, and color intensity reflects statistical significance (p-value); (**D**) Gene Ontology enrichment network graph of the comparison between WHO Grade 2 and Grade 1 gliomas. GO terms are represented as nodes interconnected by edges that denote functional similarity or gene set overlap. Solid lines represent direct functional or hierarchical relationships, while dotted lines indicate weaker or indirect associations. Edge width reflects the strength of association, with thicker lines indicating a higher degree of similarity.

**Fig. 4:**
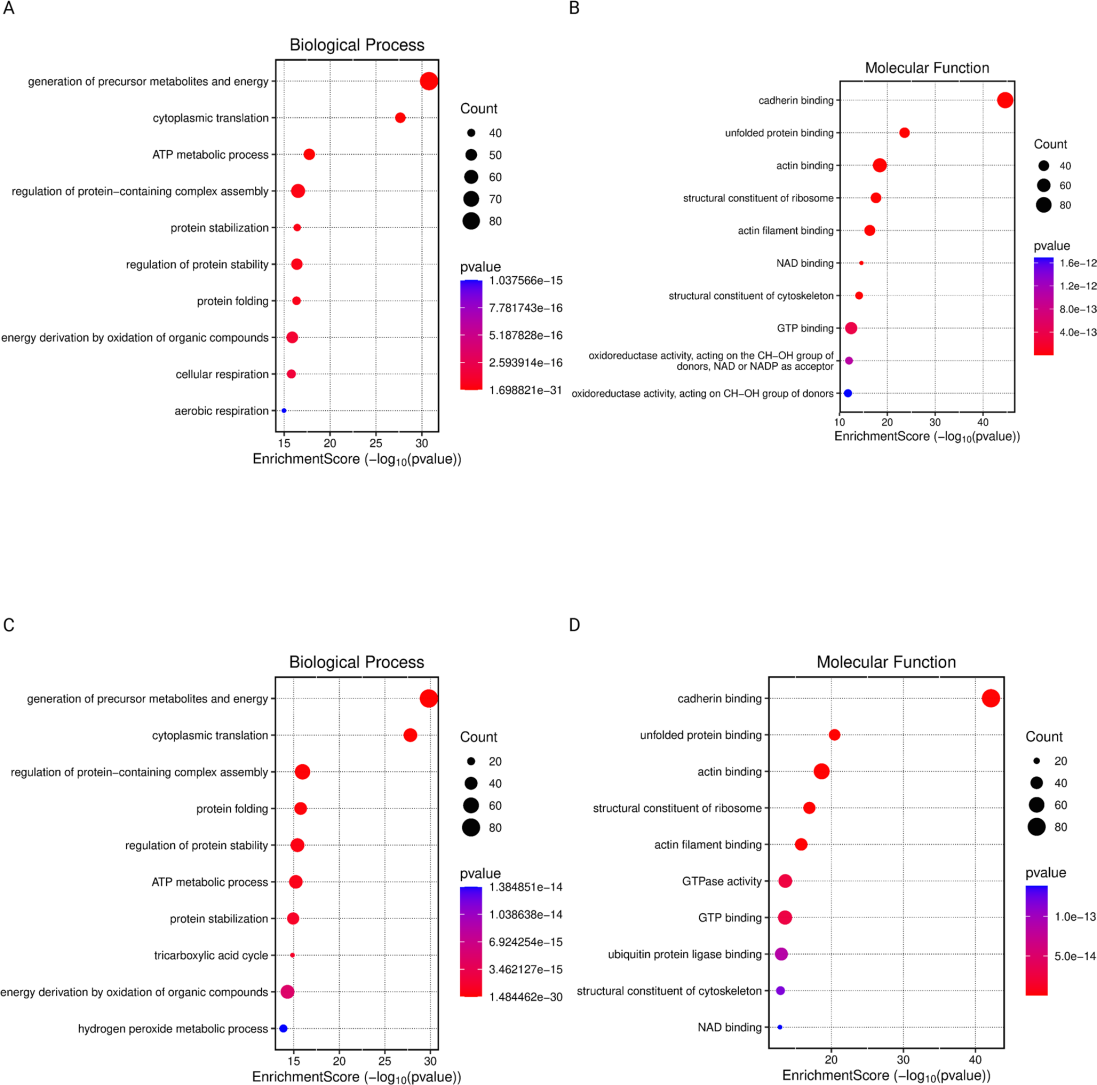
Gene Ontology-Based analysis of Biological Processes and Molecular Functions Comparing WHO Grade 3 vs. Grade 2 and WHO Grade 2 vs. Grade 1 Gliomas. (**A**) Dot plot of enriched GO Biological Processes comparing WHO Grade 3 vs. Grade 2 gliomas. Dot size represents the number of genes associated with each term, and color intensity reflects statistical significance (p-value); (**B**) Dot plot of enriched GO Molecular Functions comparing WHO Grade 3 vs. Grade 2 gliomas. Dot size represents the number of genes associated with each term, and color intensity reflects statistical significance (p-value); (**C**) Dot plot of enriched GO Biological Processes comparing WHO Grade 2 vs. Grade 1 gliomas. Dot size represents the number of genes associated with each term, and color intensity reflects statistical significance (p-value); (**D**) Dot plot of enriched GO Molecular Functions comparing WHO Grade 2 vs. Grade 1 gliomas. Dot size represents the number of genes associated with each term, and color intensity reflects statistical significance (p-value).

Comparisons between G3 and G2 (Figure 3AB) (Figure 4AB), as well as G2 and G1 (Figure 3CD) (Figure 4AB), revealed continuous and progressive alterations in biological processes, with the most significantly affected pathways related to the generation of precursor metabolites and energy, as well as cytoplasmic translation. In terms of cellular component enrichment, both comparisons highlighted changes in focal adhesion and cell-substrate junctions. Notably, alterations in cell-substrate junctions, secretory lumens, and vesicle lumens were consistent with those observed in higher-grade tumors. Additionally, the most significantly enriched molecular function in both comparisons was cadherin binding, suggesting ongoing modulation of cell-cell adhesion mechanisms across tumor progression.

### Vesicle trafficking alterations across glioma grades

#### Grade I → II transition

During the transition from Grade I to Grade II gliomas, there was a coordinated upregulation of exocytic machinery. Synaptotagmin-1 (SYT1) and Syntaxin-binding protein 1 (STXBP1), key mediators of Ca²⁺-dependent vesicle fusion, were increased (Log2FC: +0.586 and + 0.621, respectively), alongside the dopamine transporter SLC6A3 (+ 0.684), indicating enhanced synaptic-like release. Clathrin heavy chain (CLTC) also showed a moderate increase (+ 0.427), potentially reflecting baseline endocytic cycling. In contrast, Dynamin-2 (DNM2), which mediates slower constitutive endocytosis, was markedly downregulated (− 0.775), suggesting reduced internalization and a bias toward secretion (S. Figure 3A, S. Table 9).

#### Grade II → III transition

Progression to Grade III gliomas was marked by a reversal of this secretory phenotype. SYT1 expression declined (− 0.667), as did NSF (− 0.491) and STXBP2 (− 0.523), indicating a downshift in regulated exocytosis. At the same time, V-ATPase components declined, pointing to reduced vesicle acidification. Interestingly, the adaptor complex proteins showed divergent regulation: AP2A1 was downregulated (− 0.519), while AP2B1 was upregulated (+ 0.620), possibly reflecting subunit-specific adaptation for receptor internalization. DNM1 was significantly upregulated (+ 0.329), highlighting a switch to rapid, clathrin-mediated endocytosis during this stage of accelerated growth (S. Figure 3B, S. Table 7).

#### Grade III → IV transition

In the final transition to glioblastoma, we observed partial reactivation of exocytic pathways. STXBP1 expression rebounded (+ 0.447), and although SYT1 remained suppressed relative to early stages, its decline was attenuated, suggesting a limited recovery of Ca²⁺-regulated release. DNM1 remained elevated (+ 0.329), indicating continued engagement of endocytosis, though less sharply than in Grade III. NSF levels remained low, and AP2 expression showed minimal change from the prior grade (AP2B1: +0.620; AP2A1: −0.519). These findings suggest that GBM employs a hybrid vesicular trafficking model, reactivating secretion to support invasion and intercellular signaling, while sustaining selective endocytosis to modulate surface receptor availability (S. Figure 3C, S. Table 5).

#### Grade IV IDH-WT vs. IDH-MT comparison

When comparing IDH-wildtype and IDH-mutant Grade IV gliomas, vesicle trafficking differences were modest but notable. Clathrin (CLTC: −0.453) and V-ATPase subunit ATP6V1C1 (− 0.512) were further downregulated in IDH-WT tumors, suggesting additional suppression of endocytosis and vesicular acidification. These results support the notion that IDH-WT GBMs may favor prolonged growth factor signaling by reducing internalization and lysosomal degradation, although most trafficking proteins did not differ significantly between genotypes (S. Figure 3D, S. Table 3).

The proteomic data reveal grade-dependent changes in synaptic and vesicular trafficking proteins (S. Figure  3E). Presynaptic vesicle components such as synaptotagmin (SYT1) and syntaxin (STX1B) show complex patterns: SYT1 increases in G1 vs. G2 (+ 1.325, S. Table 9) but declines in higher grades (≈–0.67 log₂ in G3 vs. G4, S. Table 5), while STX1B is slightly up in G3 vs. G4 (+ 0.723, S. Table 5) but down in G3 vs. G2 (–1.217, S. Table 7).

Functionally, SNARE-related factors show complementary trends. The SNARE disassembly ATPase NSF is down in G3 vs. G4 (mean − 0.491, S. Table 5) and drops sharply from G1 to G2 (–2.427, S. Table 9), whereas α-SNAP (NAPA) is up in G3 vs. G4 (+ 0.255, S. Table 5) but down in G2 vs. G3 (–0.785, S. Table 7). This suggests a stage-specific remodeling of SNARE recycling: early grades (G1–G2) have higher NSF and lower α-SNAP relative to intermediate grades.

Endocytic machinery components exhibit modest, non-monotonic changes. Clathrin heavy chain expression is strongly reduced in the highest grade (≈–0.54 log₂ in G3 vs. G4, S. Table 5, and − 0.90 in G4-IDH-MT vs. WT, S. Table 3) but is elevated in mid-grades (up to + 1.84 in G1 vs. G2, S. Table 9). Dynamin isoforms show only minor fluctuations (mean log₂FC ∼±0.5) across grades (S. Tables 3, 5, 7 and 9), and AP-2 subunits remain near baseline (S. Tables 5 and 7). These data imply that clathrin-mediated endocytosis is relatively attenuated in aggressive gliomas, whereas early/intermediate tumors retain more of the vesicle-coat apparatus.

Notably, the vacuolar ATPase (V-ATPase) proton pump subunits show a clear downward shift in high-grade tumors: for example, V1 subunit A (ATP6V1A) is ~–1.0 log₂ in G4-IDH-MT vs. WT (S. Table 3), and all detected ATP6V subunits trend negative from G2→G4 (S.Tables 3, 5 and 7), with only a slight rebound in G1 vs. G2 (S. Table 9).

## Discussion

### Metabolic remodeling coupled to vesicle trafficking

Gliomas are recognized as plastic tumors that exploit neuronal signaling pathways to support growth, invasion, and immune evasion [7, 8, 23]. Gliomas exploit a remarkable plasticity in vesicle trafficking to support their aggressive growth, coordinating metabolic reprogramming with secretory and endocytic pathways. Indeed, our previous metabolomic research on the same group of patients [20] confirmed some of these alterations on metabolome level– notably, significantly elevated methionine and phenylalanine alongside a marked depletion of taurine, as well as shifts in membrane lipid composition (phosphatidylcholine and sphingomyelin) and tryptophan catabolism towards kynurenine and serotonin. Methionine and kynurenine accumulation are especially pronounced in high-grade gliomas, which triggers immune evasion, and promotes tumorigenesis [24]. Glioma cells avidly concentrate methionine (up to 100-fold more than normal astrocytes) and require it for proliferation. In parallel, excess tryptophan metabolism produces kynurenine– an immunosuppressive metabolite that activates aryl hydrocarbon receptor (AhR) signaling– while diverting tryptophan from serotonin synthesis unless the enzyme tryptophan hydroxylase-1 (TPH1) is upregulated [24, 25]. Intriguingly, many high-grade gliomas do overexpress TPH1, boosting serotonin production that can in turn augment NF-κB signaling and tumor invasion [25]. Thus, the observed buildup of kynurenine and serotonin in our glioma metabolome hints at a dual strategy: kynurenine helps tumors evade immune detection, while serotonin acts as an autocrine growth promoter [25, 26]. The depletion of taurine, an abundant osmolyte and neuromodulator, further reflects altered sulfur-amino acid flux– likely shunted into glutathione/hypotaurine pathways to buffer oxidative stress [27]. Notably, gliomas also feature higher levels of choline-rich phospholipids like phosphatidylcholine (PC) and sphingomyelin (SM) compared to normal brain tissue [20]. This remodeling of membrane lipids is consistent with hightened membrane turnover and vesicle biogenesis, given tumor need to continually synthesize vesicular membranes for secretion and autophagy. Collectively, these metabolic and lipidomic adaptations supply both the building blocks and signals for an aggressive phenotype– providing ample cargo (e.g. glutamate, cytokines) and flexible membranes for an upregulated vesicle trafficking system.

Crucially, the mechanistic target of rapamycin (mTOR) emerges as a central integrator linking these metabolic cues to vesicle trafficking outputs. mTORC1, a master growth regulator often hyperactive in gliomas, senses amino acid abundance on lysosomal membranes and correspondingly adjusts cellular catabolic and secretory pathways. In nutrient-replete conditions (excess methionine, etc.), mTORC1 remains active on the lysosome, phosphorylating targets like TFEB and ULK1 to suppress autophagy while promoting protein and lipid synthesis [28]. This skews the balance toward secretion: rather than digesting cargo, glioma cells can divert autophagic vesicles into an unconventional secretory route, exporting factors that remodel the microenvironment [29]. Concurrently, heightened mTOR signaling can drive increased exocytosis of lysosome-related organelles [28], amplifying the release of metabolites and signaling molecules. In essence, mTOR senses the rewired metabolism (e.g. abundant methionine, lipids) and feeds forward to enhance vesicle trafficking plasticity– enabling the tumor to rapidly deploy pro-tumorigenic cargo externally and recycle membrane receptors internally. Consistently, high mTOR activity in Grade 4 gliomas has been linked to elevated activity of the cystine–glutamate antiporter xCT [30], which facilitates glutamate secretion and couples metabolic state to excitatory output.

### Vesicular trafficking reprogramming in glioma malignancy

As gliomas progress from benign Grade 1 to highly malignant Grade 4, our proteomic analyses reveal a stepwise reorganization of vesicle trafficking machinery. Early transitions favor secretory (exocytic) components, intermediate stages emphasize endocytosis, and full-blown Grade 4 gliomas reinstate fusion machinery [31]. For example, Grade 1→2 gliomas show upregulation of exocytic SNARE proteins (SYT-1, Syntaxin-1) and neurotransmitter transporters, while dynamin-dependent fission is suppressed. Conversely, Grade 2→3 tumors downregulate these secretory factors and upregulate clathrin, AP-2 adaptors, and dynamin, indicating a shift toward endocytic pathways. By Grade 3→4, Syntaxin and dynamin levels rise again and the fusion-promoting α -SNAP is strongly activated, whereas clathrin and V-ATPase components decline. This grade-dependent pattern is summarized in Fig. 3.

Functionally, these proteomic changes suggest glioma cells initially enhance synaptic-like secretion during early transformation. The increase in Syntaxin-1 and SYT1 from Grade I to II– modest by activity scores (Syntaxin) but clear in expression– implies more vesicle docking and Ca^2+^-triggered release [32, 33]. Such secretory reprogramming could facilitate release of growth factors or modulators in the tumor microenvironment. In support, SNARE-mediated exocytosis is known to drive glioblastoma growth and invasion; experimental blockade of Syntaxin-1 greatly reduces Grade 4 glioma proliferation and invasiveness [33]. The concomitant downregulation of dynamin and neutral V-ATPase activity at this stage may reflect reduced membrane scission and acidification, skewing the balance toward exocytosis.

However, this trend reverses as tumors become more aggressive. In the Grade 2→3 transition, virtually all exocytic regulators (SYT1, Syntaxin-1, Munc18, neurotransmitter transporters) are predicted inhibited, while endocytic players (dynamin, clathrin, AP-2 adaptors) are strongly activated. Proteomically, this corresponds to an upsurge of clathrin and AP-2 in Grade 3, with SNARE factors sharply diminished. Such a switch echoes recent findings that high-grade gliomas suppress broad endocytic machinery: a proteomics study reported large-scale depletion of clathrin, AP-2, dynamins and related factors in glioma, leading to sustained receptor signaling [34]. Our data refine this view temporally, suggesting that early transformation (Grade 1→2) initially sidesteps endocytosis, but by Grade 3 gliomas have mobilized clathrin-mediated uptake– perhaps to internalize growth factor receptors for recycling or downregulation. This dynamic remodeling may help explain the oncogenic necessity of fine-tuning RTK signaling; impaired endocytosis indeed has been proposed to prolong RTK activity in glioma [34].

With full malignancy (Grade 3→4), glioma cells appear to re-engage exocytic fusion. Syntaxin-1 and dynamin rebound in expression and activity, suggesting renewed emphasis on vesicle fusion and fission [33, 34]. Notably, α-SNAP (a SNARE disassembly factor) is strongly activated in Grade 4, indicating robust SNARE complex cycling for fusion events. In contrast, clathrin is now neutral or slightly inhibited, and V-ATPase subunits are downregulated [35]. Thus, glioblastoma may rely on a mixed strategy: it restores secretory SNARE machinery (potentially to support invasive secretion and cell–cell communication) while dialing back bulk endocytosis [36–38]. The decline in clathrin/AP-2 at this late stage is consistent with an overall shutdown of endocytic infrastructure in Grade 4. Indeed, our proteomics align with those prior reports: most high-grade gliomas maintain lower levels of clathrin and adaptins relative to lower grades [34].

Importantly, these grade-wise shifts also bear on IDH mutation status in Grade 4. IDH-wildtype Grade 4 tumors show a pronounced inhibition of clathrin and V-ATPase relative to IDH-MT ones. In other words, the most aggressive IDH-WT gliomas have the lowest expression of endocytic machinery and proton pump components [39]. This fits with observations that specific V-ATPase subunits predict IDH-WT glioma aggressiveness [40]. Our data suggest that IDH-WT tumors further silence clathrin-dependent trafficking, perhaps to maximize persistence of surface growth signals, and downregulate vesicle acidification. Paradoxically, however, the literature shows invasive tumors often display increased plasma-membrane V-ATPase for acidifying the microenvironment [39, 40]. This discrepancy could reflect subunit switching or altered trafficking not captured at the gross expression level; nonetheless, it underscores that our proteomic changes likely denote reprogramming of acidification rather than simple loss of function.

Interpreting specific components, we note isoform-dependent dynamics of dynamin. Dynamin-1 (DNM1) and dynamin-2 (DNM2) are both cancer-promoting GTPases [41], yet they behave differently here. DNM1 expression peaks in Grade II and again in Grade IV, whereas DNM2 falls in Grade II then rebounds by Grade III. This may reflect their known tissue contexts: DNM1 is neuron-enriched and supplies rapid vesicle scission, while ubiquitous DNM2 mediates slower endocytosis [41]. Possibly, early-stage gliomas upregulate DNM1 to support synaptic-like vesicle recycling, then transition to DNM2-mediated uptake in intermediate stages. Later, DNM1 reappears in GBM to drive aggressive endocytic turnover when needed. Both isoforms being oncogenic, their oscillating profiles highlight glioma plasticity: the tumor can swap isoforms to tune membrane dynamics as it evolves [41, 42].

SNARE and synaptotagmin trajectories likewise imply functional adaptation. Syntaxin-1 gradually increases through higher grades, consistent with its role in tumor proliferation and invasion [33]. SYT1, however, surges only in the intermediate Grade 2 then plummets by Grade 3 before partially recovering [43]. This suggests that Ca^2+^-triggered exocytosis is engaged transiently during early malignancy, then suppressed as tumors possibly switch to more constitutive secretion, and reactivated at end-stage. The observed decline of V-ATPase with grade also implies altered synaptic vesicle acidification– perhaps high-grade gliomas rely less on pH-driven loading of vesicles and more on other secretory routes [44–46].

Overall, our findings paint a picture of progressive remodeling of vesicle trafficking programs during glioma progression. At each step, the balance between exocytosis and endocytosis is returned, likely to meet evolving demands of growth factor signaling, migration, and microenvironment interaction. Early gliomas acquire neuron-like secretory machinery (a form of “neuronal mimicry”) to possibly communicate with their environment. As malignancy increases, these cells diminish synaptic machinery and instead amplify endocytic uptake, which may sustain oncogenic signaling and metabolic adaptation. Finally, full glioblastomas incorporate both high fusion capacity and selective endocytosis, equipping them with a versatile membrane trafficking network. This stepwise reprogramming may underpin glioma plasticity– for instance, the ability of tumor cells to form functional synapses with neurons and exploit activity-dependent cues [7]. By charting these vesicle trafficking shifts, we highlight novel aspects of how glioma cells dynamically co-opt neuronal trafficking pathways to drive malignancy.

## Conclusions

These observations illustrate that gliomas (especially higher grades) are not static glial tumors but highly plastic, quasi-neuronal entities. They can transiently adopt or drop neuronal characteristics (like synaptic protein expression or transmitter handling) to suit their needs at different stages. Early on, integrating into the brain signaling milieu may help the tumor establish itself. Later, aggressive growth might take precedence, but ultimately the tumor re-engages neuronal interactions to maximize invasion and support. This plasticity complicates treatment– targeting one mechanism (e.g., blocking a growth factor) may be insufficient if the tumor can switch to alternate pathways (like direct synaptic input). Nonetheless, the identification of these neuronal mechanisms opens new avenues: for example, blocking neuron-glioma synaptic transmission (using AMPA receptor antagonists or neuroligin inhibitors) and inhibiting key vesicle proteins (like syntaxin or synaptotagmins) to slow tumor progression.

In summary, changes in vesicle trafficking and synaptic machinery proteins across grades demonstrate how glioma cells evolve from relatively passive cells into aggressive, neuron-mimicking invaders. Upregulation of SYT1 and syntaxin in lower grades may presage the tumor engagement with neural circuits, while their fluctuations and the consistent downregulation of V-ATPase and neurotransmitter transporters in higher grades reflect a shift toward a more malignant strategy– one that forsakes normal homeostasis in favor of a hostile, pro-tumor environment. These adaptations underscore the remarkable ability of gliomas to co-opt neuronal systems for invasion, communication, and survival, ultimately highlighting tumor plasticity as both a hallmark of astrocytoma progression and a potential Achilles’ heel to exploit in future therapies.

## Electronic supplementary material

Below is the link to the electronic supplementary material.

S. Tables

S. Figures 1 and 2

S. Figure 3

## Data Availability

The datasets generated during and/or analyzed during the current study are available from the corresponding author on request.

## Abbreviations

AP2: Adaptor Protein Complex 2
BCA: Bicinchoninic Acid
CNS: Central Nervous System
FCN1: Ficolin-1
FDR: False Discovery Rate
FASP: Filter-Aided Sample Preparation
GBM: Glioblastoma
GO: Gene Ontology
HPLC: High-Performance Liquid Chromatography
H_2_O: Water
IDH-WT: Isocitrate Dehydrogenase Wild-Type
IDH-MT: Isocitrate Dehydrogenase Mutant
IPA: Ingenuity Pathway Analysis
IS: Internal Standard
KEGG: Kyoto Encyclopedia of Genes and Genomes
LC-MS: Liquid Chromatography–Mass Spectrometry
mTOR: Mechanistic Target of Rapamycin
PC: Phosphatidylcholine
PEAKS: Protein Expression Analysis and Knowledge System
SM: Sphingomyelin
SYT: Synaptotagmin
SDC: Sodium Deoxycholate
SNAP: Soluble NSF Attachment Protein
SNARE: Soluble NSF Attachment Protein Receptor
TCA: Tricarboxylic Acid
TEAB: Triethylammonium Bicarbonate
TFA: Trifluoroacetic Acid
TMT: Tandem Mass Tag
TCEP: Tris (2-carboxyethyl) phosphine
V-ATPase: Vesicular ATPase
WHO: World Health Organization
