# Supplementary material for: Proteomic profiling reveals dynamic regulation of vesicle trafficking across glioma grades
Source: J Neurooncol. 2025 Jul 24;175(2):585–98. doi: 10.1007/s11060-025-05151-5 (PMC12420692; doi:10.1007/s11060-025-05151-5)
Supplement: Supplementary file 2 — S. Figures 1 and 2 [file 11060_2025_5151_MOESM2_ESM.docx]

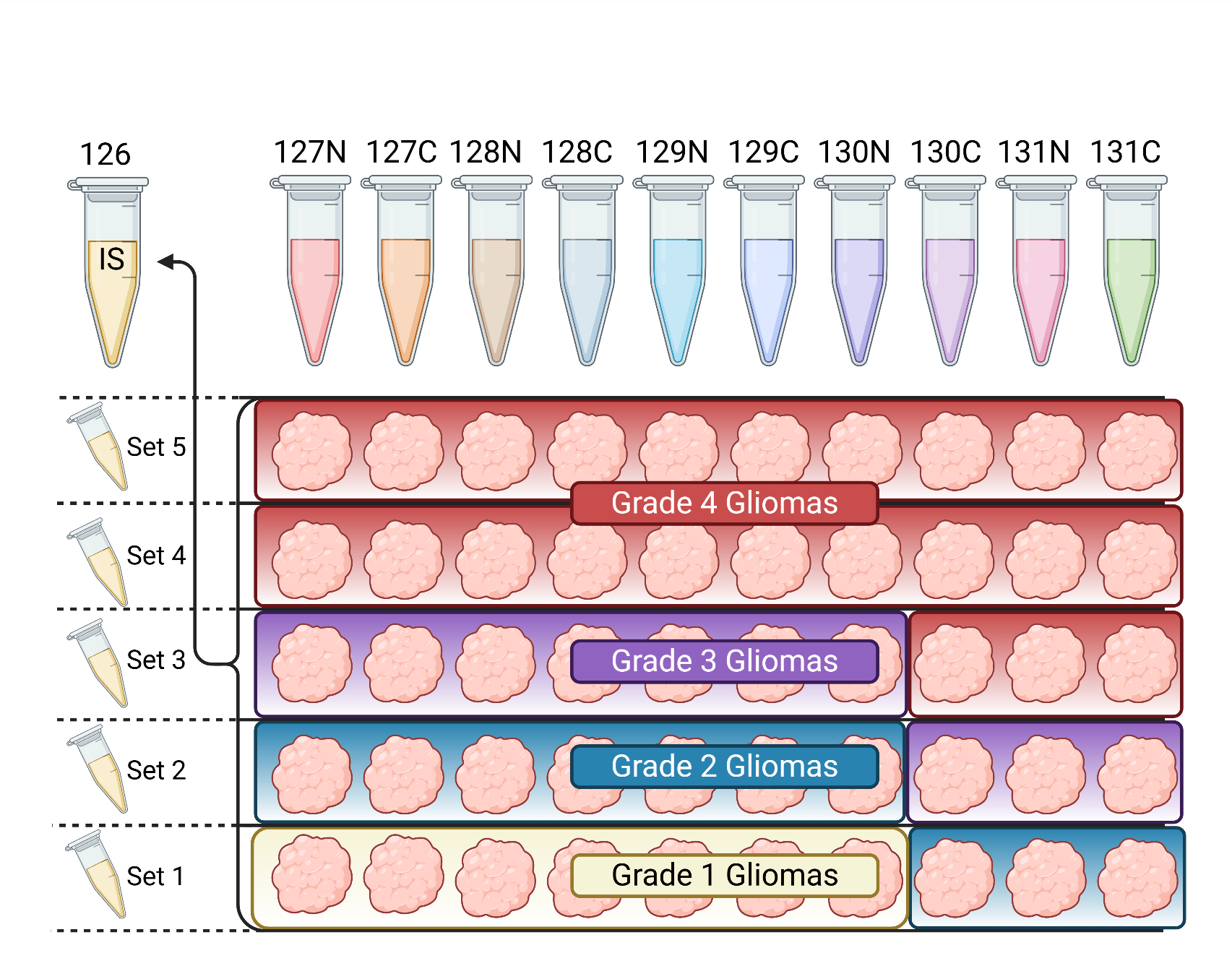


**S.Figure 1. Overview of experimental design for TMT-labelled glioma sample analysis.** In total, 2,532, 2,378, 2,645, 2,036, and 2,155 proteins were identified in TMT Sets 1 through 5, respectively, without applying any missing value filtering. These values represent protein identifications within individual 11plex batches and are lower than the totals reported for group-wise comparisons (e.g., 4,444 for G4-WT or 4,400 for G3), which reflect merged data across multiple sets. The higher counts in group-level analyses result from combining biological replicates, allowing proteins quantified in only a subset of samples to be retained in the final comparison matrices.


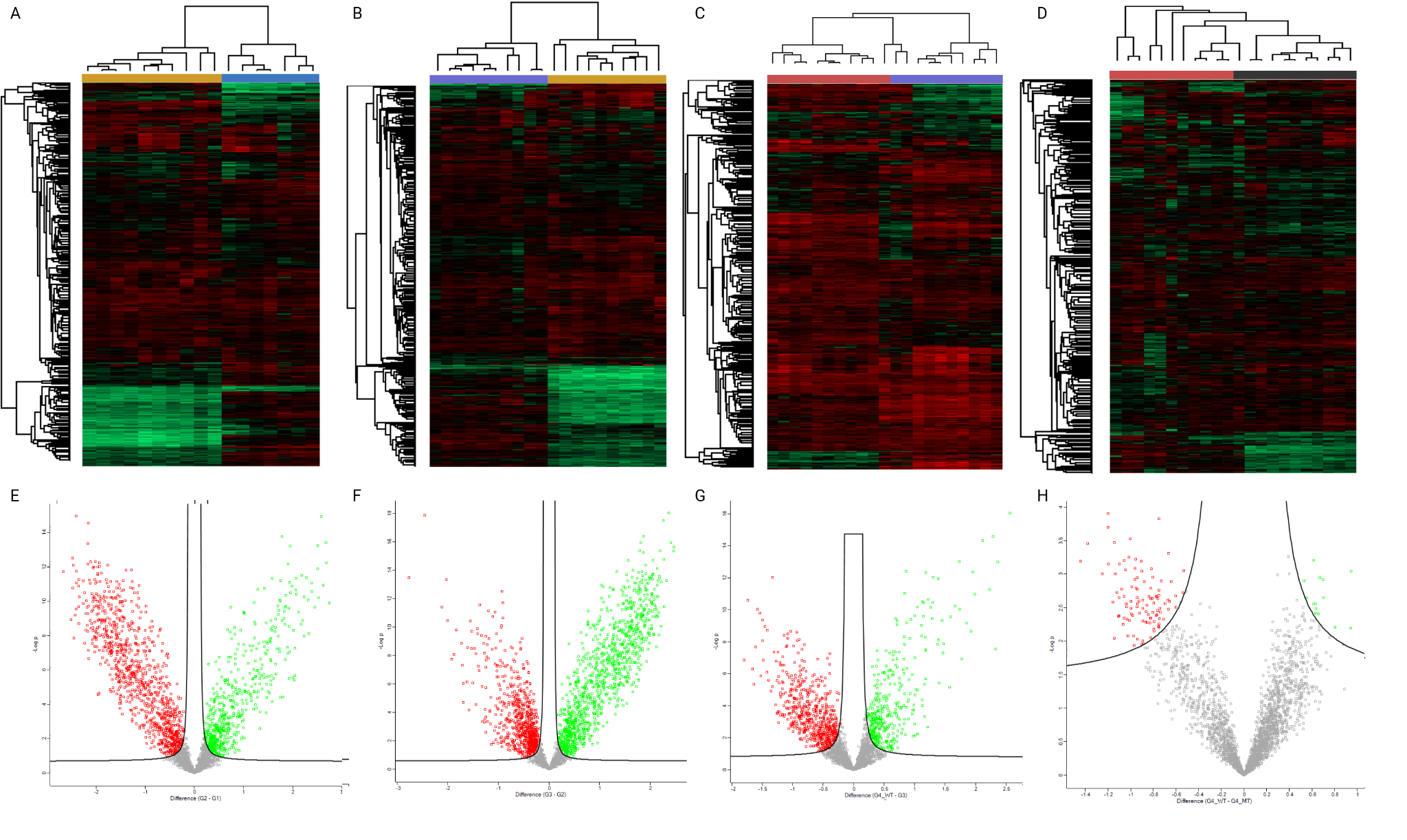


**S.Figure 2 A heatmaps of proteins identified in glioma samples**; A, WHO Grade 2 (orange) compared to WHO Grade 1 (blue); B, WHO Grade 3 (purple) compared to WHO Grade 2 (orange); C, WHO Grade 4 IDH-WT (red) compared to WHO Grade 3 (purple); D, WHO Grade 4 IDH-WT (red) compared to WHO Grade 4 IDH-MT; E, Scatter plot of WHO Grade 2 to 1 comparison; F, Scatter plot of WHO Grade 3 to 2 comparison; G, , Scatter plot of WHO Grade 4 IDH-WT to 3 comparison; H, , Scatter plot of WHO Grade 4 IDH-WT to 4 IDH-MT comparison.
