# Supplementary material for: Proteomic profiling reveals dynamic regulation of vesicle trafficking across glioma grades
Source: J Neurooncol. 2025 Jul 24;175(2):585–98. doi: 10.1007/s11060-025-05151-5 (PMC12420692; doi:10.1007/s11060-025-05151-5)
Supplement: Supplementary file 3 — S. Figure 3 [file 11060_2025_5151_MOESM3_ESM.zip › caption.docx]

**S. Figure 3. Schematic diagram of the KEGG Pathways of Synaptic Vesicle Cycle between comparisons.** A, Differences between WHO Grade 2 to WHO Grade 1 Gliomas; B, Differences between WHO Grade 3 to WHO Grade 2 Gliomas; C, Differences between WHO Grade 4 IDH-WT to WHO Grade 3 Gliomas; D, Differences between WHO Grade 4 IDH-WT to WHO Grade 4 IDH-MT Gliomas. Green indicates decreased protein levels (pathway inhibition), gray indicates no significant difference between groups, and red indicates increased protein levels (pathway activation); E, Temporal trajectories of vesicle trafficking protein expression across glioma progression; Line plot shows the mean log₂ fold changes for vesicle trafficking protein categories across glioma grade comparisons: G1 vs G2, G2 vs G3, G3 vs G4, and G4 IDH-WT vs IDH-MT. Values represent the average of all detected isoforms or subunits per category, including Synaptotagmin (*SYT1*), Syntaxin (*STXBP1, STXBP2*), NSF (*N-ethylmaleimide-sensitive factor*), Dynamin (*DNM1, DNM2*), Clathrin (*CLTC*), AP-2 complex (*AP2A1, AP2B1*), α-SNAP (*NAPA*), V-ATPase subunits (*ATP6V1C1, ATP6V1A, ATP6V0D1*), and neurotransmitter transporters (*SLC6A3, SLC1A2, SLC1A3*). Notable trends include early upregulation of exocytic machinery and neurotransmitter transporters (G1–G2), suppression of SNARE and vesicle acidification components in intermediate grades (G2–G3), and late-stage downregulation of clathrin-mediated endocytosis and proton pump subunits in high-grade gliomas. Mean values were calculated by averaging log₂ fold changes across all proteins detected within each category per comparison. Categories with no detected proteins in a given contrast were excluded from that time point.
