# Supplementary figures and images for: Proteomic profiling reveals dynamic regulation of vesicle trafficking across glioma grades
Source: J Neurooncol. 2025 Jul 24;175(2):585–98. doi: 10.1007/s11060-025-05151-5 (PMC12420692; doi:10.1007/s11060-025-05151-5)

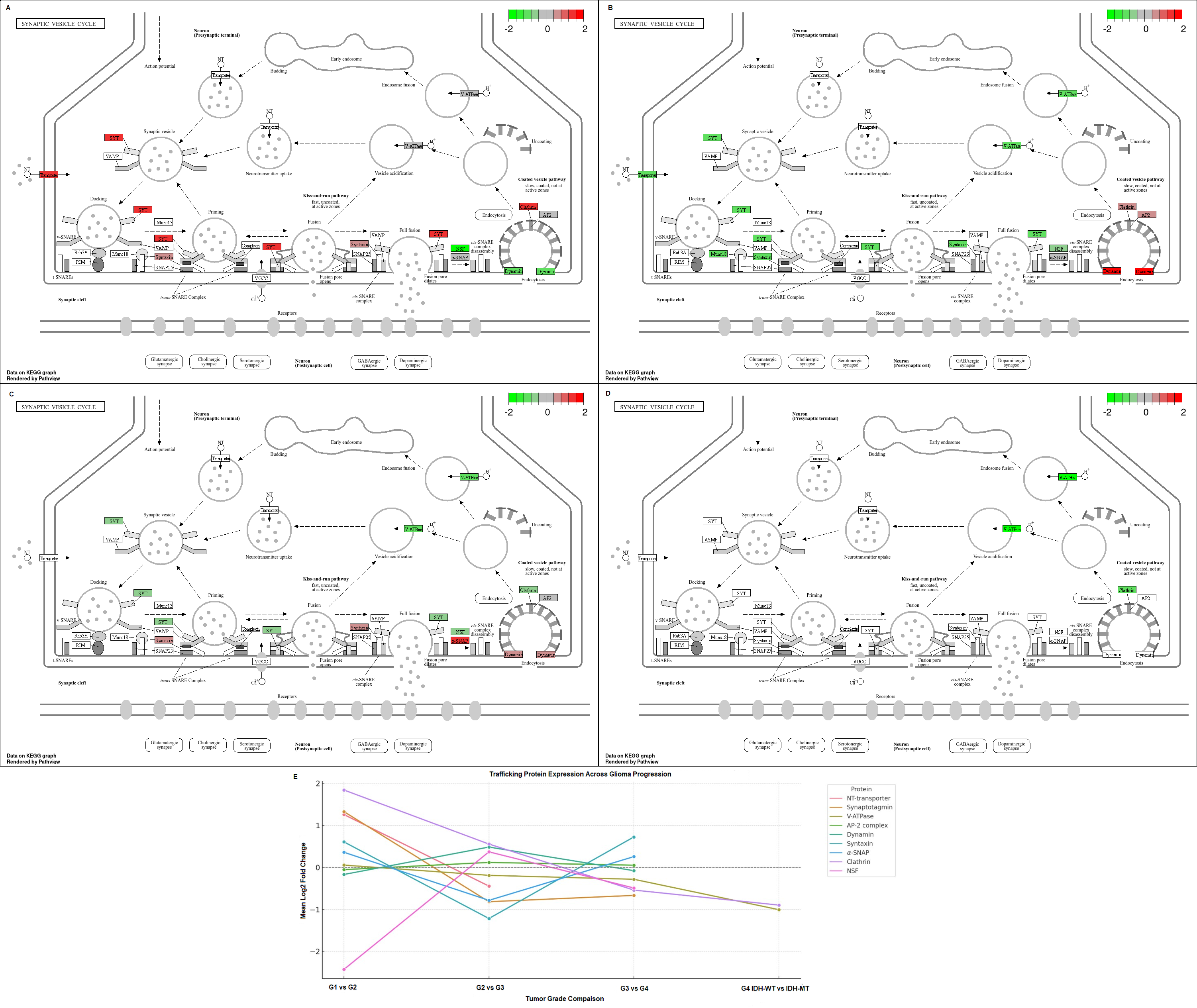

Supplement: Supplementary file 3 — S. Figure 3 [file 11060_2025_5151_MOESM3_ESM.zip › Combined_Panels.tiff]

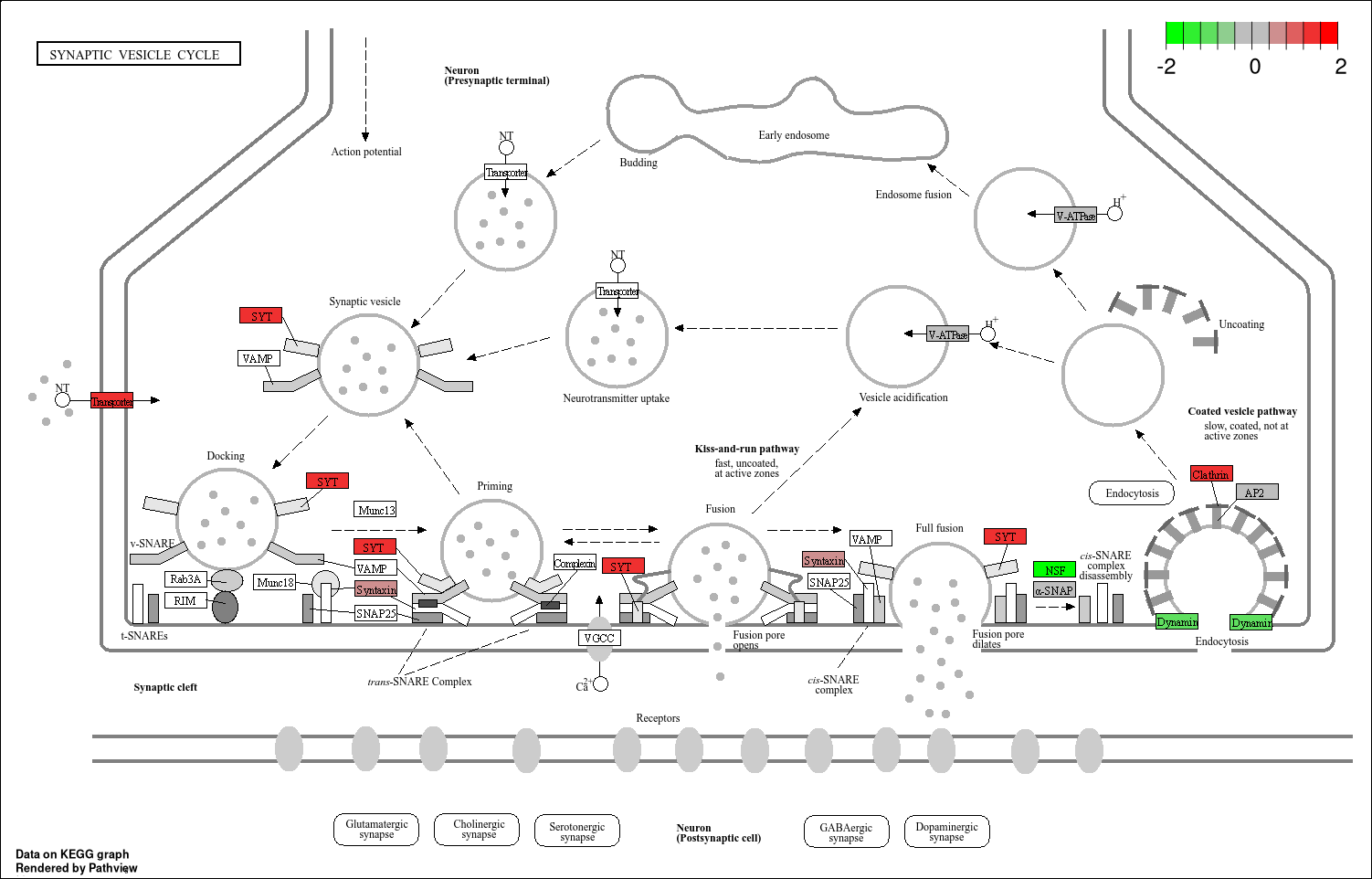

Supplement: Supplementary file 3 — S. Figure 3 [file 11060_2025_5151_MOESM3_ESM.zip › Panel A.tiff]

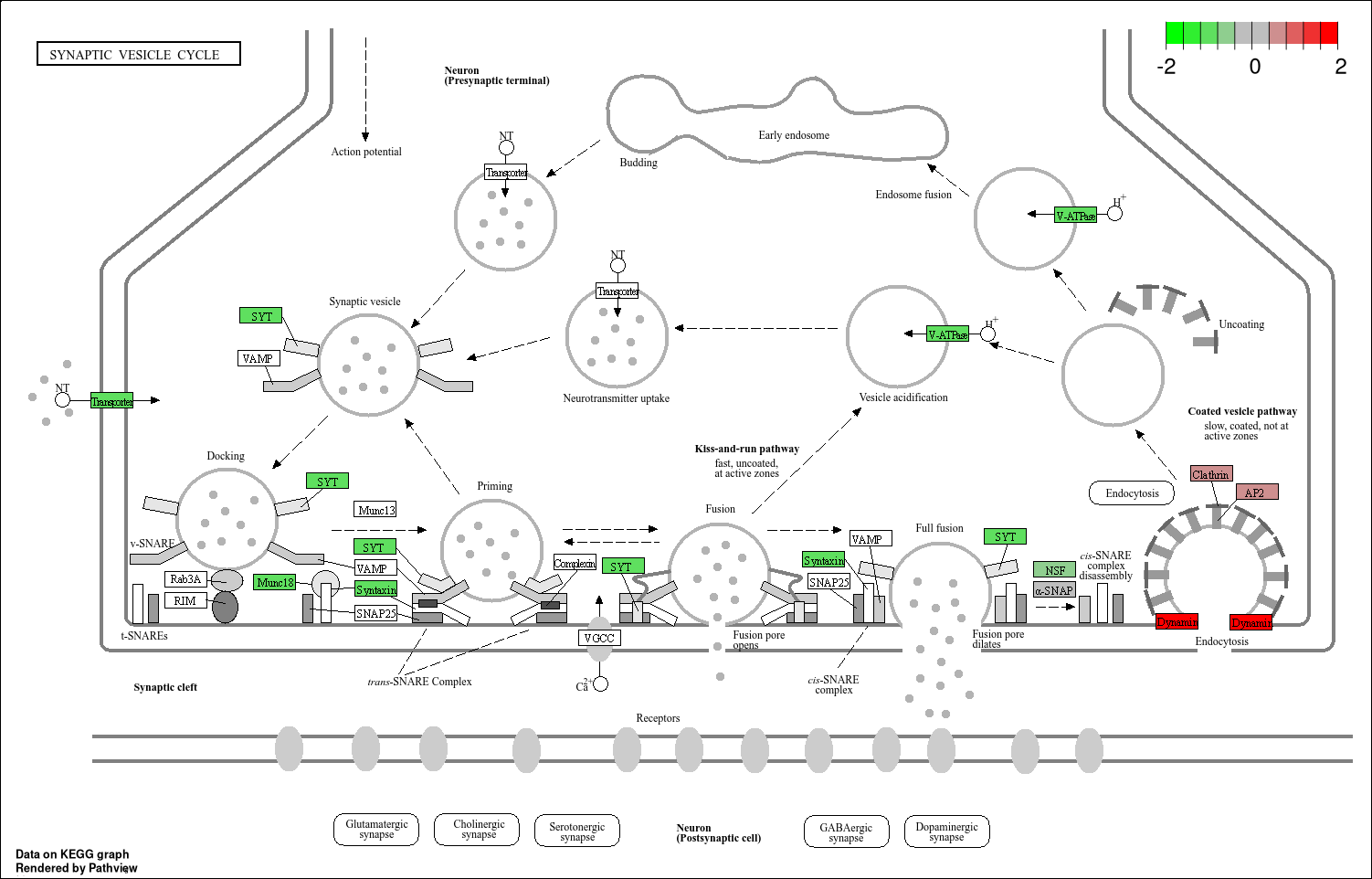

Supplement: Supplementary file 3 — S. Figure 3 [file 11060_2025_5151_MOESM3_ESM.zip › Panel B.tiff]

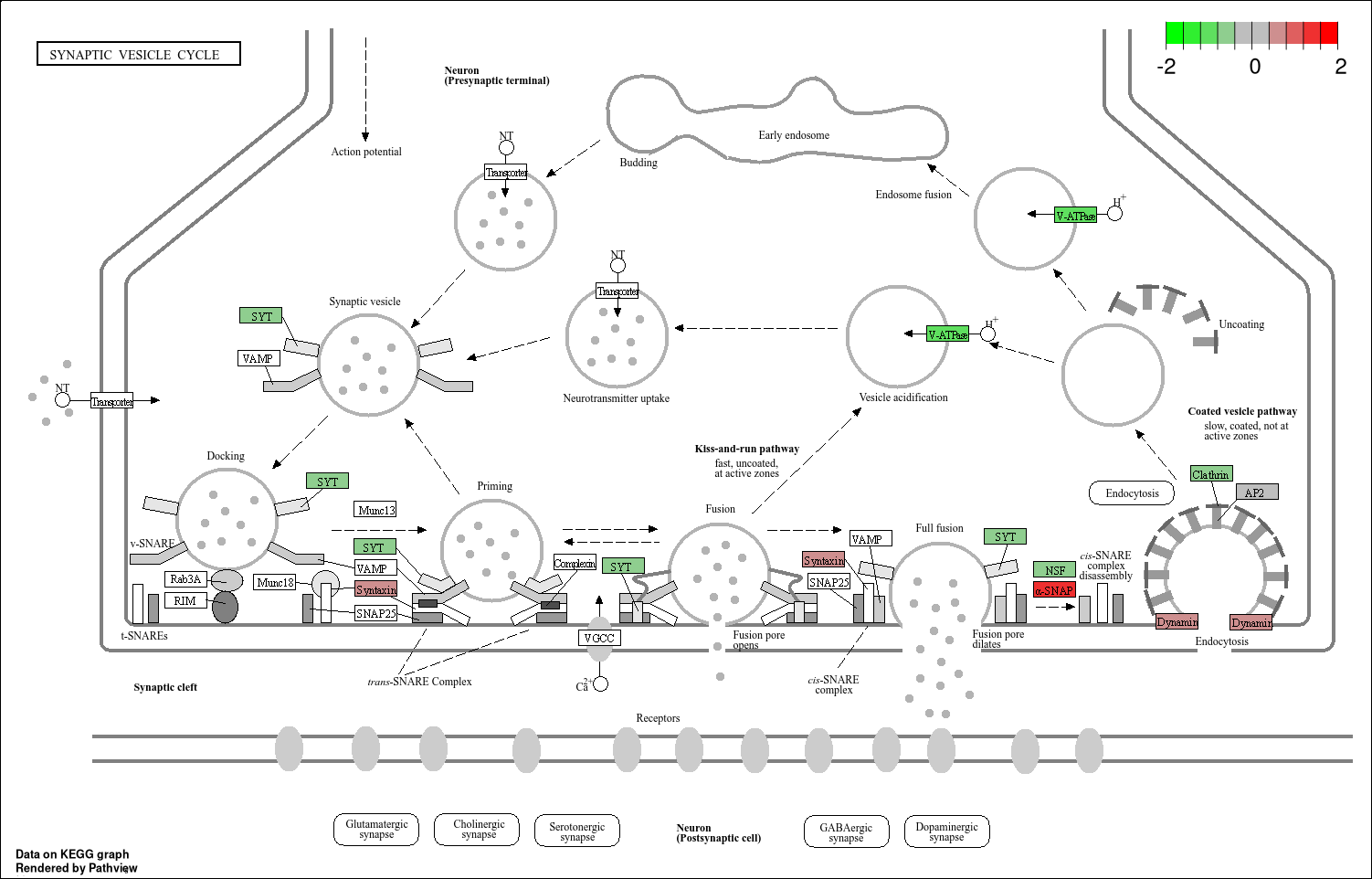

Supplement: Supplementary file 3 — S. Figure 3 [file 11060_2025_5151_MOESM3_ESM.zip › Panel C.tiff]

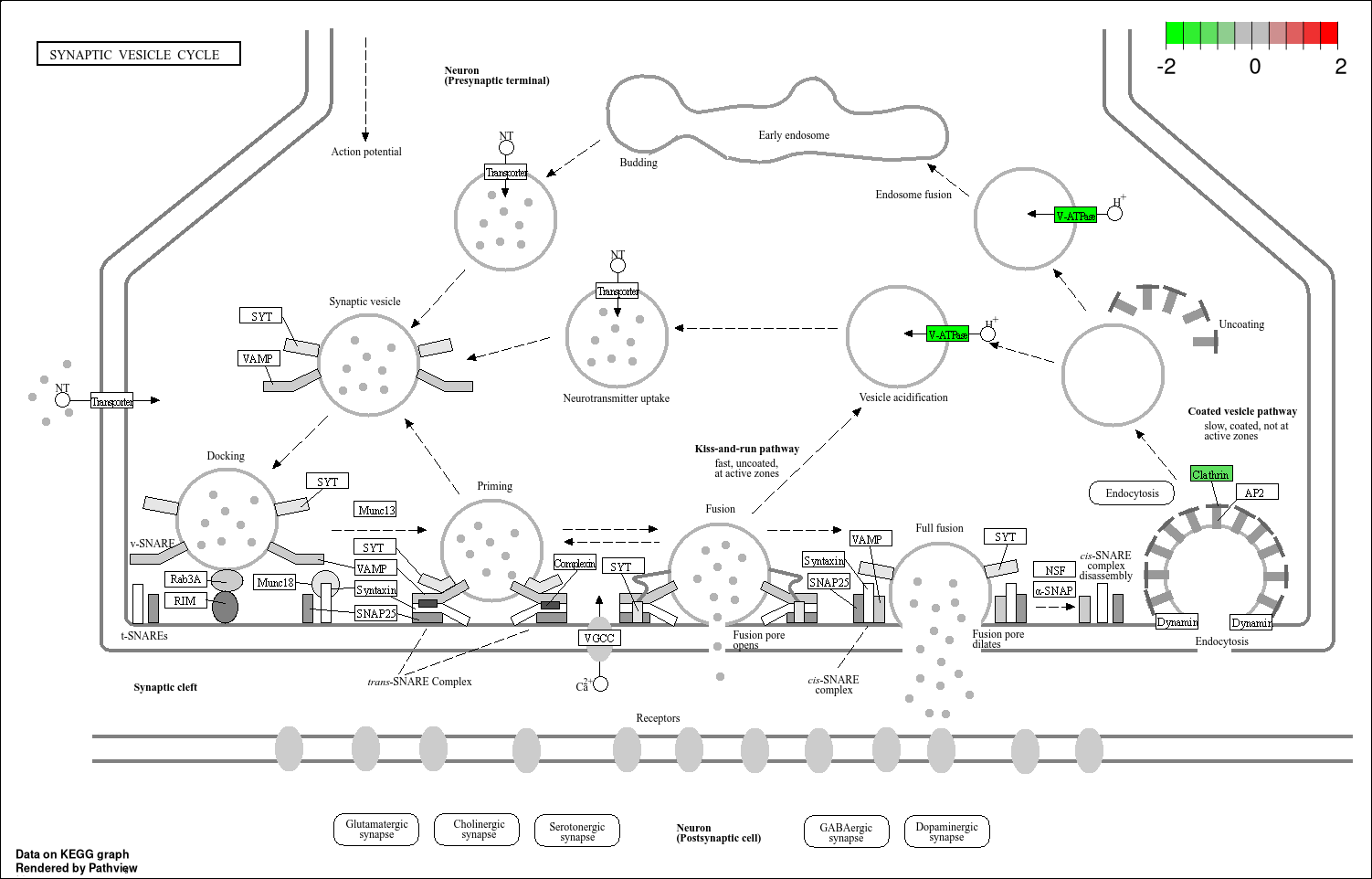

Supplement: Supplementary file 3 — S. Figure 3 [file 11060_2025_5151_MOESM3_ESM.zip › Panel D.tiff]

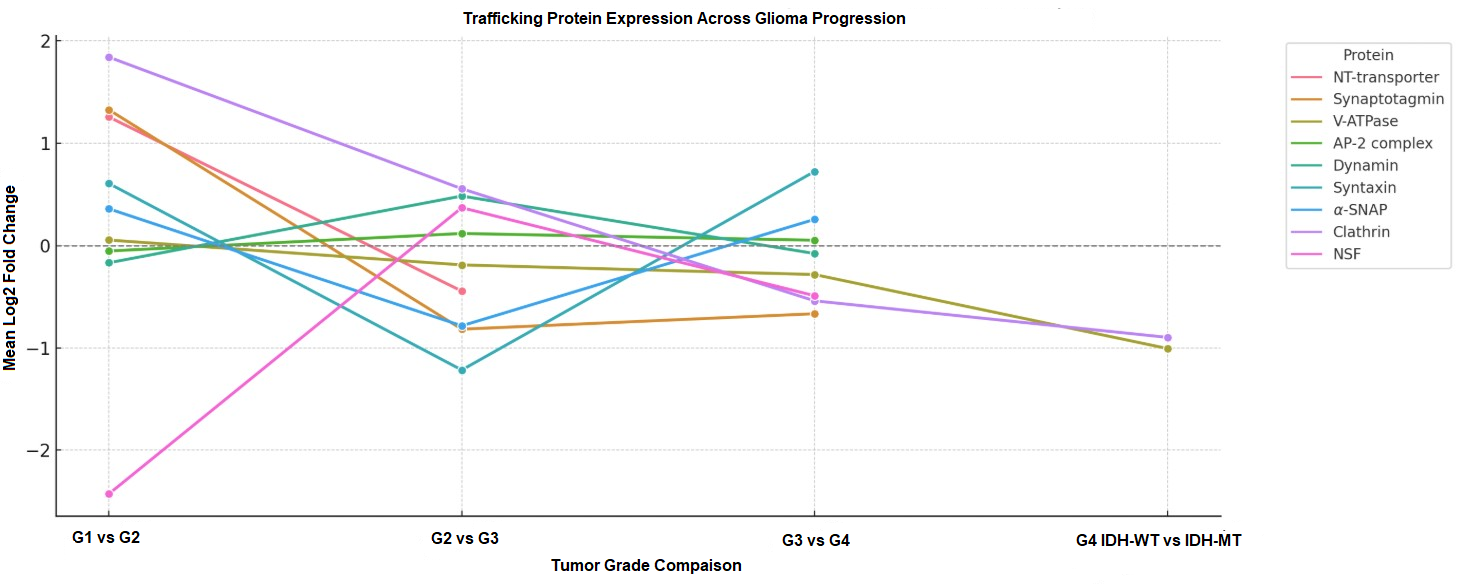

Supplement: Supplementary file 3 — S. Figure 3 [file 11060_2025_5151_MOESM3_ESM.zip › Panel E.tiff]
